# Pro-inflammatory State in Monoclonal Gammopathy of Undetermined Significance and in Multiple Myeloma Is Characterized by Low Sialylation of Pathogen-Specific and Other Monoclonal Immunoglobulins

**DOI:** 10.3389/fimmu.2017.01347

**Published:** 2017-10-19

**Authors:** Adrien Bosseboeuf, Sophie Allain-Maillet, Nicolas Mennesson, Anne Tallet, Cédric Rossi, Laurent Garderet, Denis Caillot, Philippe Moreau, Eric Piver, François Girodon, Hélène Perreault, Sophie Brouard, Arnaud Nicot, Edith Bigot-Corbel, Sylvie Hermouet, Jean Harb

**Affiliations:** ^1^CRCINA, INSERM, Institut de Recherche en Santé 2 (IRS-2), Université de Nantes, Nantes, France; ^2^Laboratoire de Biochimie, Centre Hospitalier Universitaire de Tours, Tours, France; ^3^Clinical Hematology, Centre Hospitalier Universitaire De Dijon, Dijon, France; ^4^UMRS938, INSERM Institut National de la Santé et de la Recherche Médicale, Paris, France; ^5^Département d’Hématologie et de Thérapie Cellulaire, Hôpital Saint Antoine, Paris, France; ^6^UPMC Université Paris 6, Sorbonne Universités, Paris, France; ^7^Hematology Department, Centre Hospitalier Universitaire (CHU) de Nantes, Nantes, France; ^8^UMR966, INSERM Institut National de la Santé et de la Recherche Médicale, Tours, France; ^9^Laboratoire d’Hématologie, Centre Hospitalier Universitaire De Dijon, Dijon, France; ^10^Department of Chemistry, University of Manitoba, Winnipeg, MB, Canada; ^11^Centre de Recherche en Transplantation et Immunologie UMR1064, INSERM Institut National de la Santé et de la Recherche Médicale, Université de Nantes, Nantes, France; ^12^Laboratoire de Biochimie, Centre Hospitalier Universitaire (CHU) de Nantes, Nantes, France; ^13^Faculté de Pharmacie, Université de Nantes, Nantes, France; ^14^Faculté de Médecine, Université de Nantes, Nantes, France; ^15^Laboratoire d’Hématologie, Centre Hospitalier Universitaire (CHU) de Nantes, Nantes, France

**Keywords:** myeloma, monoclonal gammopathy of undetermined significance, monoclonal immunoglobulin, immunoglobulin G sialylation, infection, inflammation, cytokines

## Abstract

Multiple myeloma (MM) and its pre-cancerous stage monoclonal gammopathy of undetermined significance (MGUS) allow to study immune responses and the chronology of inflammation in the context of blood malignancies. Both diseases are characterized by the production of a monoclonal immunoglobulin (mc Ig) which for subsets of MGUS and MM patients targets pathogens known to cause latent infection, a major cause of inflammation. Inflammation may influence the structure of both polyclonal (pc) Ig and mc Ig produced by malignant plasma cells *via* the sialylation of Ig Fc fragment. Here, we characterized the sialylation of purified mc and pc IgGs from 148 MGUS and MM patients, in comparison to pc IgGs from 46 healthy volunteers. The inflammatory state of patients was assessed by the quantification in serum of 40 inflammation-linked cytokines, using Luminex technology. While pc IgGs from MGUS and MM patients showed heterogeneity in sialylation level, mc IgGs from both MGUS and MM patients exhibited a very low level of sialylation. Furthermore, mc IgGs from MM patients were less sialylated than mc IgGs from MGUS patients (*p* < 0.01), and mc IgGs found to target an infectious pathogen showed a lower level of sialylation than mc IgGs of undetermined specificity (*p* = 0.048). Regarding inflammation, 14 cytokines were similarly elevated with a *p* value < 0.0001 in MGUS and in MM compared to healthy controls. MM differed from MGUS by higher levels of HGF, IL-11, RANTES and SDF-1-α (*p* < 0.05). MGUS and MM patients presenting with hyposialylated pc IgGs had significantly higher levels of HGF, IL-6, tumor necrosis factor-α, TGF-β1, IL-17, and IL-33 compared to patients with hyper-sialylated pc IgGs (*p* < 0.05). In MGUS and in MM, the degree of sialylation of mc and pc IgGs and the levels of four cytokines important for the anti-microbial response were correlated, either positively (IFN-α2, IL-13) or negatively (IL-17, IL-33). Thus in MGUS as in MM, hyposialylation of mc IgGs is concomitant with increased levels of cytokines that play a major role in inflammation and anti-microbial response, which implies that infection, inflammation, and abnormal immune response contribute to the pathogenesis of MGUS and MM.

## Introduction

Infectious pathogens are implicated in various B-cell malignancies (Burkitt, Hodgkin, and non-Hodgkin lymphoma, chronic lymphocytic leukemia) *via* cell infection and direct transformation [Epstein–Barr virus (EBV), hepatitis C virus (HCV)], or *via* antigen (Ag)-driven stimulation and indirect cell transformation (*Helicobacter pylori*), or both (1–5). Chronic cancer-associated inflammation is established in hematological malignancies, especially in myeloma and chronic myeloproliferative neoplasms (MPNs). Myeloma is characterized by the accumulation of malignant, clonal, mature plasma cells, which produce a monoclonal immunoglobulin (mc Ig): Ig G, A, or more rarely, M, D, and E. In multiple myeloma (MM), the quantity of mc Ig is ≥30 g/L and, thus, represents the majority of Ig measured in blood serum, typically ≥90% of IgGs; thus, most patients still produce polyclonal (non-malignant) IgGs, at low levels. Myeloma derives from a chronic stage called monoclonal gammopathy of undetermined significance (MGUS) (6). In MGUS the quantity of mc Ig in blood is <30 g/L, and the mc Ig may represent 20–70% of all IgGs; thus in MGUS, the production of polyclonal (pc) IgG is maintained, though frequently reduced compared to healthy individuals. Most MGUS never evolve toward smoldering myeloma (SM) and MM: the risk of transformation of MGUS into SM and MM is estimated at 1% per year per patient, and involves the repeated acquisition of genetic alterations (7, 8). Clonal plasma cells also depend on certain inflammation cytokines for their growth [for instance, interleukin 6 (IL-6)]. Inflammation-linked cytokines produced at high levels by malignant hematopoietic cells in myeloma and in other blood malignancies include hepatocyte growth factor (HGF), IL-11, IL-6, and IL-8 (9). Clonal myeloma cells also secrete factors that inhibit the growth of normal hematopoietic progenitors and suppress the formation of polyclonal Ig [tumor growth factor β_1_ (TGF-β_1_) and stroma cell-derived factor 1α (SDF-1α)].

Inflammatory cytokines are produced in large quantity in chronic hematological malignancies but the reasons remain unclear, and are likely multiple. Some cytokines may be produced by malignant cells as a consequence of genetic alterations (IL-6), but there is strong evidence that cytokines are also produced independently from gene mutations or re-arrangements, both by clonal and non-clonal cells (9, 10). Latent infection is a plausible cause of chronic overproduction of inflammation cytokines by various cell types. A promising approach to understand hematological malignancy is that for subsets of patients, abnormal immune response to infection by lymphoid or myeloid cells leads to chronic Ag-driven cell proliferation, polyclonal at first, then oligoclonal, and finally monoclonal. Over time, the chronically stimulated lineage is at the origin of an increased risk of genetic alteration leading to clonality and malignant transformation. To support this pathogenic process in MGUS and myeloma, we recently reported that six infectious pathogens, including carcinogenic viruses [EBV, HCV, Herpes simplex virus (HSV)] and bacteria (*H. pylori*), are the targets of ~23% of purified mc IgG from MGUS, SM, and MM patients (11).

In MGUS and MM, chronic inflammation may directly influence the structure and function of the mc IgG produced by the clonal plasma cells. Moreover, IgG molecules can trigger pro- or anti-inflammatory responses mediated by their crystallizable (Fc) fragment domain. Numerous studies provided evidence that carbohydrates attached to the IgG Fc domain are essential for IgG function (12). In fact, IgG Fc contains a single, highly conserved asparagine 297 (N297) glycosylation site, and anti-inflammatory activities of IgG have been associated with the presence of sialic acid. Thus, patients with autoimmune diseases such as rheumatoid arthritis show low levels of IgG Fc sialylation (13, 14). Inversely, IgG sialylation increases during pregnancy, and increased IgG sialylation is associated with the remission of rheumatoid arthritis (15). Moreover, the anti-inflammatory activity of intra-venous (i.v.) immunoglobulin (IVIg) injection in various murine models is due to their sialylated Fc fragments (16–19). Similarly, anti-gp120 antibodies (Abs) in HIV patients are less galactosylated and sialylated in long-term non progressors, who are infected but asymptomatic, compared to infected patients who shows disease symptoms (20).

The pro- or anti-inflammatory effector functions of IgG subclass Abs are mediated by their different affinities for activating FcγRs (FcγRI, RIIa, RIIIa, and RIIIb) and inhibiting FcγRIIb expressed by immune cells (21–23). Several studies demonstrated that the high level of sialylation on the IgG Fc fragment decreases their Ab-dependent cell-mediated cytotoxicity (ADCC) potential through less affinity for activating receptors (24). In the context of autoimmune diseases, Kaneko et al. demonstrated that the sialylated Fc fragment of IVIg was effective in treating arthritis in a mouse model (18). Similar results were obtained using four independent *in vivo* models under preventive as well as therapeutic conditions (25). The underlying mechanisms involve SIGN-R1 in mice and DC-SIGN lectin in humans; these molecules are expressed at the surface of regulatory macrophages and bind sialylated IgGs (19, 26). Subsequently, IL-33 and IL-4 cytokines modulate the inhibitory FcγRIIb receptors expressed by effector macrophages present at the site of inflammation, raising their activation threshold (26, 27). Recently, Quast et al. (28) demonstrated that tetra sialylation of IgG Fc domain impairs complement-dependent cytotoxicity (CDC) but has no impact on ADCC. This effect is due to decreased binding of C1q to Fc-galactosylated IgG. This tetra Fc-sialylated form has been demonstrated to enhance anti-inflammatory activity up to 10-fold more than IVIg across different animal models (29). Conversely, bisecting *N*-acetylglucosamines are pro-inflammatory and enhance ADCC (30). The removal of core fucose residues selectively enhances the affinity of IgG for FcγIIIa receptors, leading to an increased ADCC and decreased CDC (31). Hence, Ab that cause fetal or neonatal alloimmune thrombocytopenia have a decreased IgG1-Fc fucosylation and an increased affinity for FcγRIIIa/b receptors (32). It was demonstrated in the context of allergy that the induction of tolerance for T cell-dependent (33) or T cell-independent Ag (34) produces regulatory sialylated IgGs in mice and in humans. Tolerance was linked to an increase in α2,6-sialyltransferase in plasma cells (33). However, Jones et al. (35) recently demonstrated that IgG sialylation was B-cell-independent and that sialylation also occur in the bloodstream due to the action of a liver-secreted α2,6-sialyltransferase and the presence of platelet α-granule-derived CMP-sialic acid. Thus, this model enables a rapid functional shift in existing IgG, independent of *de novo* synthesis or recycling.

In MGUS and MM, the glycosylation state of mc IgGs has rarely been studied. While Fleming et al. (36) showed higher sialylation of IgGs from MM patients compared to MGUS patients, Nishiura et al. (37) reported less galactosylated IgGs and, consequently, hyposialylated IgGs, in MM patients compared to MGUS patients and healthy volunteers (HVs). Similarly Mittermayr et al. (38) recently described a decrease of IgG sialylation in a few MM patients in comparison to MGUS patients. In the three studies, the authors studied the glycosylation of all IgG together, without separating mc IgG from polyclonal (pc) IgGs.

Here, we report on MGUS and MM with mc IgG, the most frequent type of mc Ig in both MGUS (70–75% cases) and MM (60% cases): after purification of mc IgGs and pc IgGs from patients, the degree of sialylation of each category of IgGs was determined, and the different levels of IgG sialylation were analyzed in relation with the inflammation status of patients and the infectious pathogen targeted by the purified mc IgG.

## Materials and Methods

### Patients and Ethics Statement

The study was performed with the approval of the local ethics committee (# RC12 0085, University Hospital of Nantes) and the Commission Nationale de l’Informatique et des Libertés (CNIL # 912335). For technical reasons, only mc IgG could be purified, thus only patients presenting with a mc IgG were included in this study. Thus, we examined 148 patients with mc IgG: 68 MGUS, 6 SM, and 74 MM diagnosed at the French University Hospitals (CHU) of Dijon, Nantes, Paris (Saint-Antoine) and Tours. In this study, all MGUS patients had a mc IgG ≥4 g/L. Sera from 46 HVs and 40 patients diagnosed with MPN were also studied as controls. Written informed consents were obtained from patients in the relevant clinical departments, and in the blood bank for HVs enrolled by the Etablissement Français du Sang (EFS, Nantes, France). A convention has been signed between our laboratory (CRTI—INSERM UMR 1064) and the blood bank (EFS Pays de La Loire).

### Purification of Monoclonal and Polyclonal IgG

After clotting, blood samples of patients were centrifuged at 2,200 × *g* for 15 min at 4°C, serum was collected, and aliquots were stored at −80°C or −20°C, depending on the collecting site. Total IgG concentration in serum was measured with an immuno-nephelemetric assay performed on a Beckman Immage Analyzer (Beckman Coulter, Villepinte, France). The concentration of the monoclonal (component) IgG is estimated by integrating the electrophoretic peak according to the orthogonal mode (the so-called “baseline method”). Purification of pc and mc IgGs and verification of their purity were performed as described (1, 3, 11, 39). Briefly, after separation using electric charge on agarose gel electrophoresis (SAS-MX high resolution, Helena Biosciences, Gateshead, UK), bands corresponding to mc IgG or gamma zone corresponding to pc IgGs were carefully cut and proteins were eluted from gels into PBS. Concentration of the purified pc and mc IgGs was determined using the Nanodrop Spectro-photometer ND-1000 with the IgG extinction coefficient (ε = 1.36 for a solution of 1 mg/mL). The recovered IgG amount after purification varied from 40 to 70% for both mc IgG and pc IgG, depending on experiments and the initial IgG concentration in serum. Purity of each IgG fraction was analyzed by isoelectrophoresis and immunoblotting (homemade isoelectrofocusing gel using a range of pH 3–10, blotting onto PVDF membrane and revelation using an HRP anti-human IgG gamma chain). Only highly purified mc IgG were used for sialylation studies (see Figure S1 in Supplementary Material).

### Analysis of IgG Sialylation

An enzyme-linked lectin assay (ELLA) was developed and used for IgG sialylation detection, and an enzyme-linked immunosorbent assay (ELISA) was developed for total IgG detection, as described (40). Ninety-six well plates (NuncMaxiSorp™) were coated overnight at 4°C with 50 µL of affinipure donkey anti-human IgG, Fcγ-specific fragment Ab (Jackson ImmunoResearch, West Grove, PA, USA) diluted at 1/250 (5.2 µg/mL, ELLA) and 1/1,000 (1.3 µg/mL, ELISA) in 25 mM borate buffer pH9. After 3 washes with 200 µL PBS-Tween 0.05% (Sigma, St. Louis, MO, USA), 100 µL periodic acid (5 mM) per well were added for 10 min at room temperature (RT), protected from light. The plates were then saturated with 100 µL of B-grade bovine gelatin (Sigma, St. Louis, MO, USA) 0.25% in PBS-Tween 0.01%, at 37°C, for 2 h. After three washes, samples were diluted in PBS-Tween 0.1% and deposited in triplicates containing 1.25 ng/well for detection of total IgG, or 100 ng/well for sialylation detection. Total IgG quantity was revealed by incubating the plates with 50 µL of peroxidase affinipure donkey anti-human IgG (H + L) diluted 1/1,000 (0.8 µg/mL, Jackson ImmunoResearch, West Grove, PA, USA) for 1 h. Sialic acid was revealed using 50 µL biotinylated *Sambucus nigra* agglutinin (SNA) diluted 1/750 (2 µg/mL, Glycodiag, Orleans, France) for 90 min and then 50 µL streptavidin HRP diluted 1/1,000 (1 µg/mL, Vector laboratories, Burlingame, CA, USA) for 1 h, at 37°C. Then 50 µL of TMB, the chromogenic substrate for HRP (Sigma-Aldrich, St. Louis, MO, USA) was added and the reaction was stopped by 50 µL sulfuric acid 0.5 M after 5 min for IgG detection, and after 15 min for sialic acid detection. Optical densities (OD) were measured using Spark 10 M multimode microplate reader (Tecan, Männedorf, Switzerland) at 450 nm. The relative sialylation was expressed as the sialic acid/IgG OD ratio. Control samples were used in all experimental settings, to assess reproducibility.

### Isoelectrophoretic Studies

A 1% agarose gel containing a 10% mixture of 3–10 and 8–10.5 ampholytes was prepared and pre-focalized with acetic acid (0.5 N) and sodium hydroxide (1 N) in order to establish a pH gradient. The pre-focalization was run for 30 min at 250 V and 30 mA for a total of 90 vH. Samples were then dropped at the anode side and the focalization was launched during 90 min at 1,200 V and 50 mA for a total of 900 vH. Sera proteins were then passively transferred on a pre-activated PVDF Immobilon-P membrane (Millipore, Billenca, CA, USA) for a few minutes. Finally, after a saturation step and several washes, the membrane was incubated with the rabbit anti-human IgG (H + L)-peroxydase (Dako, Santa Clara, CA, USA). Revelation was made using HRP revelation system (Sigma, St. Louis, MO, USA).

### Mass Spectrometry

Purified IgGs were digested by trypsin and glycopeptides were isolated from peptides using two methods, i.e., reversed-phase high-performance liquid chromatography and a protocol involving the commercial ProteoExtract^®^ Glycopeptide Enrichment Kit (EMD-Millipore, Etobicoke, ON, Canada). Fractions were concentrated for analysis by matrix-assisted laser desorption/ionization time-of-flight mass spectrometry (MALDI-ToF-MS). The instrument used was an UltraFleXtreme™ (Bruker, Bremen, Germany) operated in positive ion, reflective mode.

### Quantification of Inflammation Cytokines

Frozen aliquots of serum were used to quantify 40 cytokines and 2 soluble cytokine receptors linked to inflammation or/and infection using the Luminex technology (Bio-Plex 200) with Bio-Plex Pro Human Cytokine Panel kits (Bio-Rad, Hercules, CA, USA), following the manufacturer’s instructions.

### MIAA Assay

The Multiplexed Infectious Antigen microArray (MIAA) assay has been described previously (11, 39). The assay was developed to determine the infectious specificity of purified IgG using commercially available Ag or/and lysates from EBV, HCV, cytomegalovirus (CMV), Herpes simplex virus 1 (HSV-1), HSV-2, varicella zoster virus (VZV), *Helicobacter pylori (H. pylori), Toxoplasma gondii* (*T. gondii*), and *Borrelia burgdorferi* (*B. burgdorferi)*. Infectious Ag were purchased from Abcam (Cambridge, United Kingdom), Advanced Biotechnologies Inc. (Columbia, MD, USA) and ImmunoDiag (Hämeenlinna, Finland). Lysates were supplied by Advanced Biotechnologies Inc. (Columbia, MD, USA) and EastCoast Bio (North Berwick, ME, USA). The arrays consist of 8 × 8 matrices that included: (i) 13 Ag: 2 for EBV, 3 for HCV, 1 for *T. gondii*, 1 for *H. pylori*, 2 for HSV-1, 2 for HSV-2, and 2 for VZV; (ii) 5 lysates: CMV, *T. gondii, H. pylori*, HSV-1, and HSV-2; (iii) 2 mixes: one of 5 CMV Ags and one of 2 *B. burgdorferi* Ags; (iv) 2 negative controls: PBS, and PBS with 0.1% bovine serum albumin (BSA). For hybridization, IgG concentrations were adjusted to 400 µg/mL for serum and from 50 to 200 µg/mL for purified mc IgG. 80 µL of samples were incubated for 2 h at RT. After washing, slides were incubated with a labeled secondary Ab (0.2 µg/mL DylightTM 680 Labeled Goat anti-human IgG (H + L), from SeraCare, Milford, MA, USA; Ref. 5230-0342). Fluorescence signal, detected with the Odyssey infrared imaging system scanner at 21 µm resolution (LI-COR Biosciences, NE, USA) was quantified using the GenePix^®^ Pro 4 Microarray Acquisition & Analysis Software (Molecular Devices, Sunnyvale, CA, USA).

### Statistics

Data analysis was performed by GraphPad Prism 6.01 software. Patient parameters were expressed as medians and ranges, or/and means ± SEM. The chi-2 test was used for categorical variables. For continuous variables (*n* ≥ 30) the Student *t*-test or the one-way ANOVA followed by Tukey’s *post hoc* test were used. For continuous variables (*n* < 30), a normality test was systematically performed for each group. When parametric conditions were fulfilled, a Student’s *t*-test or a one-way ANOVA followed by Tukey’s *post hoc* test was performed. For non-parametric conditions, a Mann–Whitney *U* test or a Kruskal–Wallis test followed by Dunn’s *post hoc* test was performed. The tests used are indicated in Figure and Table legends. A *P* value below 0.05 was considered statistically significant.

## Results

### Purification of mc and pc IgGs

**Figure 1:**
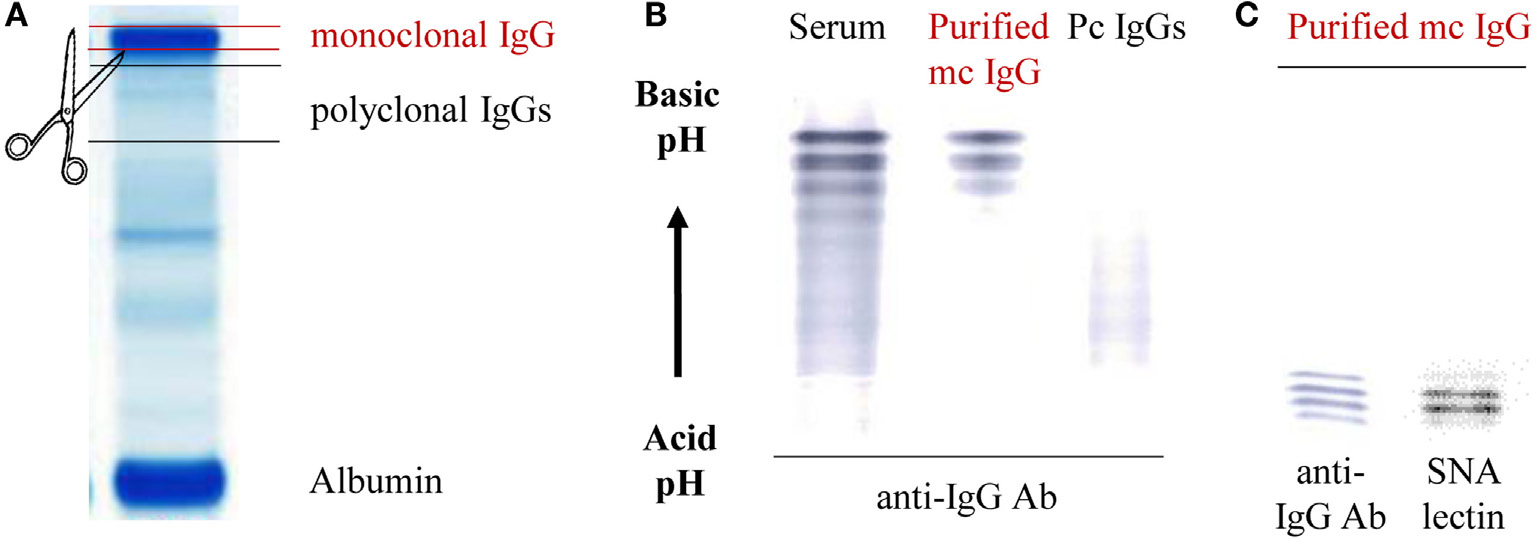
Purification scheme of mc IgGs from the serum of monoclonal gammopathy of undetermined significance (MGUS) and multiple myeloma (MM) patients. **(A)** Mc and pc IgGs from MGUS and MM patients were submitted to high-resolution agarose gel electrophoresis and then cut from the gel. **(B)** The purity of mc and pc IgGs was checked *via* isoelectro-focalization (IEF) and immunoblotting using a peroxydase coupled to an anti-human IgG-γ chain antibody (HRP anti-human IgG Ab). **(C)** The sialylation of mc IgG was assessed after incubation with biotinylated *Sambucus nigra* agglutinin (SNA) and streptavidin peroxidase. **(B,C)** are representative of purified basic or acid mc IgG, respectively.

According to the patients’ data, in the present cohort the mc IgG represented 40.8–87.7% (median: 70.8%) of total IgGs in MGUS, and 49.7–95.5% (median: 86.3%) in MM. These percentages represent estimations, since mc IgGs and total IgGs are not measured by the same techniques. However, the data indicated that almost all MGUS and MM patients in the cohort produced some level of pc IgGs, estimated to represent ~30 and ~14% (medians) of total IgGs in MGUS and in MM, respectively. After electrophoresis of the 148 sera on agarose gel, mc IgGs and pc IgGs from each patient were separated. The relevant bands were collected, and proteins were eluted from the gel (Figure 1A). The purity of IgGs was confirmed by immunoblotting after isoelectophoresis. Examples of the efficiency of this method are illustrated in Figure 1B and Figure S1 in Supplementary Material. The typical pattern of mc IgGs resolves into a series of sharp lines that are equidistant approximately 0.05 pH units apart. This difference in pH is due to the deamidation of glutaminyl and asparaginyl residues, yielding aspartic and glutamic acid, respectively (41, 42). The purified pc IgGs appear as a smear after isoelectrophoresis and are completely separated from mc IgG (Figure 1B). Only highly purified mc IgGs (*n* = 148) and pc IgGs (*n* = 142) were retained for further analysis (for 6 patients, pc IgGs could not be purified). In addition to deamidation, micro-heterogeneity of mc IgGs is due to carbohydrate differences, especially the sialylation level. Hence, immunofixation of the purified mc IgG, using biotinylated SNA, a lectin specific of sialic acid, shows that the lectin recognized some of the mc IgG bands, indicating that they are not fully sialylated (Figure 1C).

### Characteristics of Patients

In this retrospective study, we analyzed 148 patients presenting with mc IgG and diagnosed with MGUS (*n* = 68), SM (*n* = 6) or MM (*n* = 74). Clinical data were available for 133/148 patients (59 MGUS, 6 SM, 68 MM). The biological and clinical characteristics of the 133 patients are shown in Table 1. The median age for MGUS, SM, and MM patients at the time of diagnosis was similar, 67.0, 67.1, and 63.6, respectively. The male/female ratio was 55.9% for MGUS, and higher for SM and MM patients: 83.3 and 60.3%, respectively (differences not significant, Chi-2 test). The International Staging System (ISS) and Durie–Salmon Staging (DSS) scores indicated that 32.7% of MM patients presented with ISS stage III at the time of diagnosis (DSS stage III: 44.6%). In MM, mc IgG were studied either at the time of diagnosis (17 patients; 23%), or during or after treatment (40 patients; 54%); for 17 patients (23%), information was not available. In addition, two categories of control sera without mc IgG were used in this study: first, a cohort of 46 HVs (with no chronic inflammation, and no hematological disease); second, a cohort of 40 patients at the time of diagnosis of MPN, a group of chronic hematological malignancies with strong associated inflammation. As expected, MPN patients showed higher leukocyte counts, a higher hemoglobin level, and higher platelet counts compared to MGUS and MM patients.

**Table 1:** Characteristics of monoclonal gammopathy of undetermined significance (MGUS), smoldering myeloma (SM), and multiple myeloma (MM) patients.

Description	HV	Myeloproliferative neoplasms	MGUS	SM	MM
Nbr of patients (total)	46	40	68	6	74
Nbr with available biological data	0	40	59	6	68
Nbr with available sialylation data for mc IgG	0	0	68	6	74
Nbr with available sialylation data for pc IgG	46	40	68	6	68
Nbr with available cytokines expression data	9	0	34	5	25
Male sex (%)	NA	54.2%	55.9%	83.3%	60.3%
Age (years)					
Median	NA	66.2	67.0	67.1	63.6
Range	NA	33.0–95.0	31.0–96.9	40.0–84.0	45.7–86.5
Mc IgG (g/L)					
Median	NA	NA	12.0	21.8	18.5
Range	NA	NA	4.0–28.9	13.5–67.0	8.5–68.0
β_2_-Microglobulin (mg/L)					
Median	ND	ND	2.5	4.9	3.1
Range	ND	ND	1.4–10.1	2.1–5.8	1.3–12.1
Leukocytes (10^9^/L)					
Median	ND	9.3	7.1	8.5	5.1
Range	ND	2.8–41.0	0.2–34.3	6.1–29.2	0.6–19.0
Hemoglobin (g/dL)					
Median	ND	15.6	13.3	12.8	10.9
Range	ND	7.9–22.4	8.7–16.2	6.9–15.5	7.3–15.5
Platelets (10^9^/L)					
Median	ND	437.5	224.0	217.5	202.0
Range	ND	67.0–1,851.0	38.0–580.0	178.0–309.0	15.0–529.0
ISS stage III (%)	ND	ND	ND	20%	32.7%
DSS stage III (%)	ND	ND	ND	0%	44.6%

*Nbr, number of patients; HV, healthy volunteers; NA, not available; ND, not determined; ISS, International Staging System; DSS, Durie–Salmon Staging*.

For MGUS, SM, and MM patients, the degree of sialylation of purified pc and mc IgGs was analyzed separately. For a significant number of patients in this cohort (35/128, or 25.7%: MGUS, *n* = 19; SM/MM, *n* = 16), our previous studies had revealed that the purified mc IgG specifically targeted an infectious pathogen (11). Table 2 shows the serological status of patients, as determined by the MIAA assay; the results of these studies reflect the Ab function of pc IgG and mc IgG analyzed together. The MIAA used here allowed testing for panels of commercially available Ag and lysates from nine infectious pathogens: EBV (HHV-4), HCV, CMV (HHV-5), HSV-1 (HHV-1), HSV-2 (HHV-2), VZV, (HHV-3), *H. pylori, T. gondii*, and *B. burgdorferi* (39). Overall, the serological status of MGUS patients was similar to that of the general population, which is consistent with the persistence of pc IgGs for most patients. As previously reported, there were lower rates of positive serology for SM/MM patients compared to MGUS patients, likely explained by lower production of pc IgGs in advanced MM disease (11). The characteristics of the 35 patients (19 MGUS and 16 SM/MM) with infectious pathogen-specific mc IgG (“MIAA+ patients”) as determined by the MIAA assay performed with purified mc IgG, are shown in Table 3. The purified mc IgG of 22 patients (12 MGUS and 10 SM/MM) specifically targeted EBV, and the EBNA-1 protein was the main target (20/22 patients) (11). The purified mc IgG from the 13 other patients specifically targeted HSV-1 (*n* = 6), CMV (*n* = 2), VZV (*n* = 2), and *H. pylori* (*n* = 3) (11). The sialylation results of mc and pc IgGs from the 35 MIAA+ patients were analyzed separately.

**Table 2:** Serological status of patients.

Pathogens	Monoclonal gammopathy of undetermined significance (*n* = 57)		Smoldering myeloma and multiple myeloma (*n* = 71)		All patients (*n* = 128)	
	Negative	Positive	Negative	Positive	Negative	Positive
Epstein–Barr virus, nbr (%)	2	55 (96.5)	17	54 (76.1)	19	109 (85.1)
Hepatitis C virus, nbr (%)	56	1 (1.8)	71	0 (0.0)	127	1 (0.8)
Cytomegalovirus, nbr (%)	25	32 (56.1)	38	33 (46.4)	63	65 (50.8)
Herpes simplex virus (HSV)-1, nbr (%)	15	42 (73.7)	24	47 (66.2)	39	89 (69.5)
HSV-2, nbr (%)	40	17 (29.8)	54	17 (23.9)	94	34 (26.6)
Varicella zoster virus, nbr (%)	30	27 (47.4)	45	26 (36.6)	75	53 (41.4)
*Helicobacter pylori*, nbr (%)	38	19 (33.3)	55	16 (22.5)	93	35 (27.3)
*Toxoplasma gondii*, nbr (%)	30	27 (47.4)	46	25 (35.2)	76	52 (40.6)
*Borrelia burgdorferi*, nbr (%)	53	4 (7)	66	5 (7.0)	116	9 (7.0)

*Sera of patients, which include both pc and mc IgG, were analyzed with the Multiplexed Infectious Antigen microArray (MIAA) assay. Results represent the number and % of patients with IgG in serum (pc IgG + mc IgG) that target the pathogens of the MIAA assay*.

*Nbr, number*.

**Table 3:** Description of patients presenting with a pathogen-specific purified mc IgG, as assessed with the Multiplexed Infectious Antigen microArray (MIAA) assay (MIAA+ patients).

Description	Monoclonal gammopathy of undetermined significance			Smoldering myeloma and multiple myeloma		
	MIAA+	MIAA−	*P*-value	MIAA+	MIAA−	*P*-value
Patients with MIAA data	19	38		16	55	
Male sex (%)	68.4%	47.3%	NS	87.5%	54.5%	0.019
Age at diagnosis (years)						
Median	66.0	68.5	NS	71.1	63.1	NS
Range	40.6–79.0	31.0–97.0		55.2–84.0	42.0–87.0	
Mc IgG (g/L)						
Median	12.1	12.0	NS	24.4	17.7	NS
Range	5.0–27.0	4.0–28.9		11.0–48.0	8.5–68.0	
β_2_-Microglobulin (mg/L)						
Median	2.3	2.7	NS	5.5	2.7	0.040
Range	1.5–9.6	1.4–10.1		2.4–11.0	1.3–12.1	
Leukocytes (10^9^/L)						
Median	6.7	7.5	NS	6.4	5.5	NS
Range	3.3–15.5	3.6–16.0		0.6–9.7	1.7–19.0	
Hemoglobin—g/dL						
Median	13.9	13.2	NS	11.3	11.0	NS
Range	10.0–16.0	8.7–16.2		8.0–15.5	7.3–15.2	
Platelets (10^9^/L)						
Median	208.5	227.5	NS	196.5	210.0	NS
Range	124.0–318.0	75.0–580.0		15.0–309.0	78.0–529.0	
ISS stage III (%)	–	–	–	53.3%	13.6%	0.0043
DSS stage III (%)	–	–	–	68.7%	38.2%	0.047

*Statistical analysis was performed using the chi-2 test for categorical variables and the Mann–Whitney U test for continuous variables. Significant differences are indicated*.

*NS, not significant; ISS, International Staging System; DSS, Durie–Salmon Staging*.

### IgG Sialylation Level

**Figure 2:**
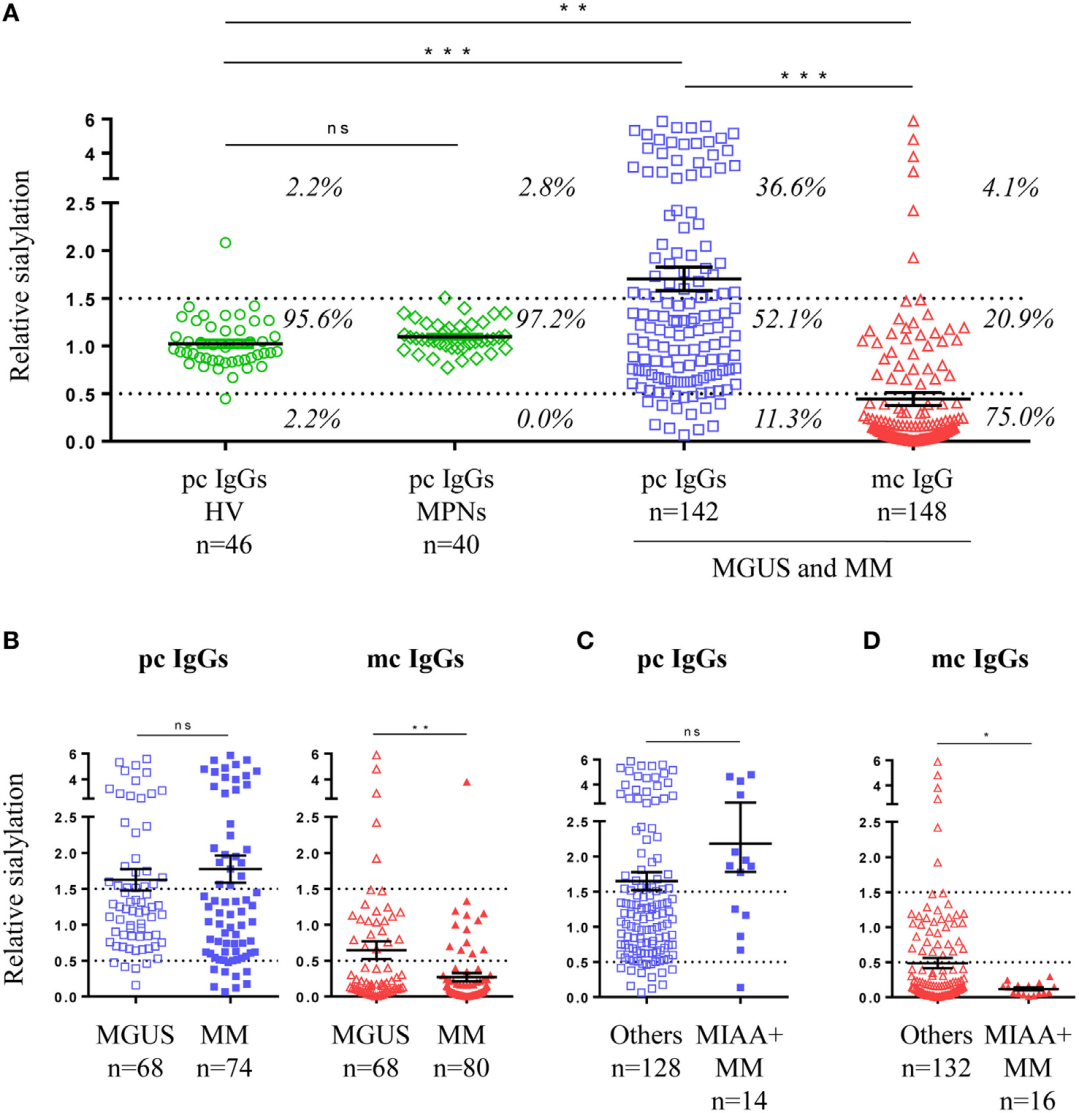
Sialylation level of IgGs in monoclonal gammopathy of undetermined significance (MGUS) and multiple myeloma (MM). **(A)** Sialylation level of purified pc IgGs from healthy volunteers (*n* = 46) and myeloproliferative neoplasms (*n* = 40), and from purified mc IgGs (*n* = 148) and pc IgGs (*n* = 142) from MGUS and SM/MM patients (for 6 patients, pc IgGs could not be purified). Percentages indicate the % of patients whose IgGs present a low (<0.5), normal (0.5–1.5), of high (>1.5) level of sialylation. **(B)** Degree of sialylation of purified pc IgGs and mc IgGs from MGUS (*n* = 68) and SM/MM (*n* = 80) patients. Sialylation level of purified pc IgGs **(C)** and mc IgGs **(D)** from MM patients with pathogen-specific mc IgG (MIAA+ patients) were then compared with pc and mc IgGs from other MM patients. Bars indicate means ± SEM. Statistical analysis was performed using one-way ANOVA test followed by Tukey’s Multiple Comparison Test for **(A)**, unpaired *t*-test for **(B)** and **(C)**, and unpaired *t*-test with Welch’s correction for **(D)**. *p < 0.05, **p < 0.01, ***p < 0.001.

The sialylation state of pc and mc IgGs is shown in Figure 2A. Analysis of the cohort of healthy controls showed that 95.6% of pc IgGs have a relative sialylation level between 0.5 and 1.5. The mean relative sialylation level of IgGs from healthy individuals was 1.023. Of note, 100% of pc IgGs samples from MPN patients had a similar relative sialylation level, between 0.5 and 1.5 (mean: 1.096). By contrast, pc IgGs from 142 MGUS, SM, and MM patients showed a large heterogeneity in relative sialylation level. For 52.1% of MGUS, SM, and MM patients, pc IgGs presented the same sialylation level as pc IgGs from healthy controls and MPN patients, but for 36.6% of MGUS, SM, and MM patients, pc IgGs were more sialylated, and for 11.3% of these patients, pc IgGs were less sialylated. Overall the degree of sialylation of pc IgGs from the MGUS/SM/MM group was significantly higher than for the group of healthy controls (mean = 1.703, *p* = 0.0007) or the group of MPN patients (*p* = 0.0062). Regarding mc IgG, for 75% of MGUS and SM/MM patients, the purified mc IgG presented with strongly reduced sialylation; only 4.1% of patients showed a high sialylation of purified mc IgG (Figure 2A). The sialylation level of purified mc IgGs from the MGUS/SM/MM group (mean = 0.443) was significantly lower than pc IgGs from the healthy controls (*p* = 0.0054), MPN patients (*p* = 0.0025), and MGUS/SM/MM patients (*p* < 0.0001) (Figure 2A). When MGUS and SM/MM were compared, the sialylation level of pc IgGs was not different, whereas that of mc IgGs was lower in SM/MM (mean = 0.271) than in MGUS (mean = 0.645, *p* = 0.0048) (Figure 2B). Regarding SM/MM patients with mc IgGs specific for a pathogen (MIAA+ patients), the sialylation level of pc IgGs (MGUS or SM/MM) was not different from other pc IgGs (Figure 2C) but pathogen-specific mc IgG were significantly less sialylated (mean = 0.117) than all other mc IgGs (mean = 0.486, *p* = 0.048) (Figure 2D).

### Relative Quantification of Sialylation Level by Mass Spectrometry

**Figure 3:**
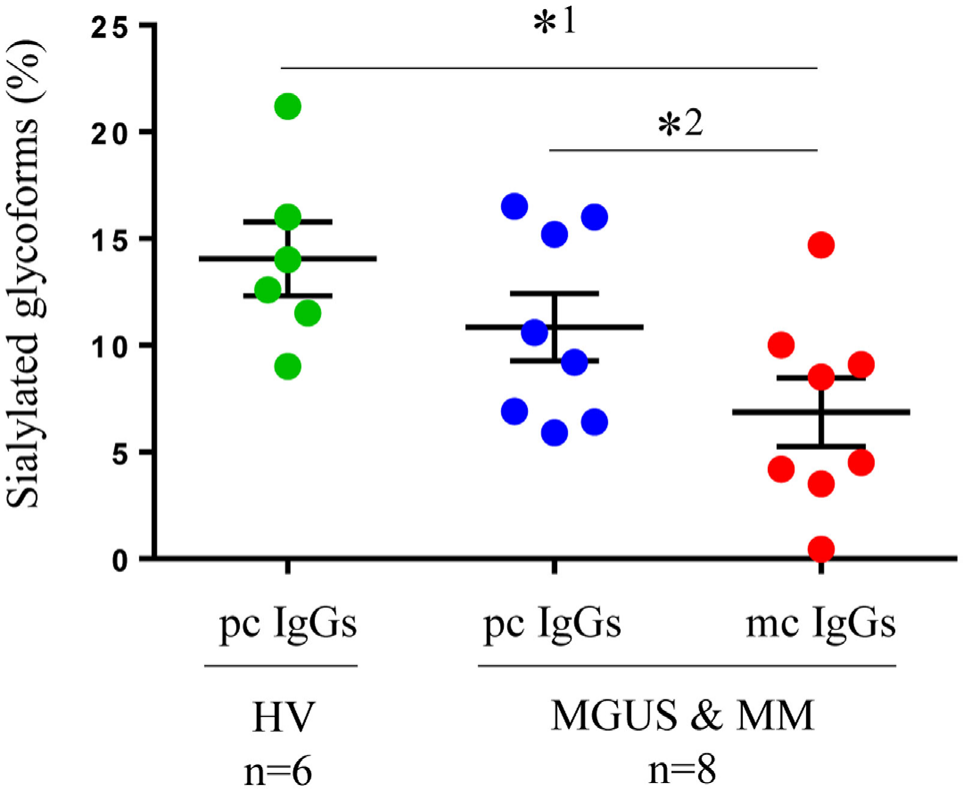
Sialylation of IgGs as assessed by mass spectrometry: Purified pc IgGs from six healthy volunteers (HV) and pc IgGs and mc IgGs from eight patients [four monoclonal gammopathy of undetermined significance (MGUS) and four multiple myeloma (MM)] were analyzed by mass spectrometry. The percentage of sialylated glycoforms (G1FS + G2FS/total peaks) was determined in pc and mc IgG fractions. Bars indicate means ± SEM. Statistical analysis was performed using one-way ANOVA test after a Kolmogorov–Smirnov normality test followed by Tukey’s comparison test (^*1^*p* < 0.05); and a paired *t*-test for pc IgG and mc IgG from MGUS and MM patients (^*2^*p* < 0.05).

In a second series of experiments, we used HPLC-mass spectrometry to confirm the differences in IgG sialylation level observed between purified mc and pc IgGs. Mass spectrometry analysis was performed on 24 samples. Figure S2 in Supplementary Material shows the results obtained for two representative purified mc IgGs that differed in their sialylation level as assessed with ELLA and ELISA. Figure S2A in Supplementary Material shows a purified mc IgG1 that was found to be highly sialylated using the ELLA and ELISA techniques. Conversely, Figure S2B in Supplementary Material shows a purified mc IgG1 found to be poorly sialylated using the ELLA and ELISA techniques. In both cases, the sialylated forms corresponding to G1FS (1 galactose, 1 fucose, and 1 sialic acid) and G2FS (two galactoses, one fucose, and one sialic acid) with a *m/z* at 3,087 and 3,249, respectively, are indicated with red arrows. The respective percentages of IgG1 in G1FS and G2FS forms were 26.4% (Figure S2A in Supplementary Material) and 5.2% (Figure S2B in Supplementary Material), thus confirming the results obtained by ELLA and ELISA (Figure 2). We then compared the results obtained for six healthy individuals and eight patients (four MGUS and four MM) (Figure 3). The sialylation level of pc IgGs from healthy controls was not different from those of pc IgGs from MGUS/MM patients. Purified mc IgGs from MGUS/MM patients were less sialylated (mean = 7.35%) than pc IgGs from the same patients (mean = 10.21%; paired *t*-test: *p* = 0.03) or from healthy controls (mean = 14.05%; *p* = 0.02), thus confirming the results obtained by ELISA (Figure 2).

### Pro-inflammatory Status of MGUS and MM Patients

For 64 patients (34 MGUS and 30 MM), we quantified in blood serum the level of 40 cytokines and 2 soluble cytokine receptors [IL-1 receptor α (IL-1Rα), IL-2Rα] linked to inflammation and, for certain molecules [interferon (IFN) α_2_, IFN-γ, eotaxin, IL-17, IL-22, IL-26, and IL-33] to anti-viral or anti-microbial immune responses (Table 4). Compared to the group of healthy controls, MGUS/SM/MM patients had significant increases in serum levels of 28 molecules: IL-1Rα, IL-2Rα, and 26 cytokines. Among those, 17 cytokines were elevated in the patients group with *p* < 0.001, compared to healthy controls: IL-6, IL-7, IL-10, IL-13, IL-15, IL-17, IL-33, IFN-α_2_, tumor necrosis factor (TNF)-α, granulocyte colony stimulating factor (G-CSF), granulocyte-macrophage CSF (GM-CSF), basic fibroblast growth factor (FGF), vascular endothelial growth factor (VEGF), monokine induced by IFN-γ (MIG, or CXCL9), monocyte chemotactic protein 1 (MCP-1 or CCL2), HGF, and leukemia inhibitory factor (LIF). Most of these cytokines are pro-inflammatory, which indicates a shift toward a pro-inflammatory environment in MGUS and SM/MM patients.

**Table 4:** Cytokine profile of healthy volunteers (HVs), monoclonal gammopathy of undetermined significance (MGUS), smoldering myeloma (SM), and multiple myeloma (MM) patients.

Cytokines	HV (*n* = 9)		MGUS and MM (*n* = 64)		MGUS and MM > HV	
	Median (pg/mL)	Range	Median (pg/mL)	Range	Fold change	*P*-value
Interleukin (IL)-4	4.50	3.61–5.15	5.65	0.12–23.89	1.26	**0.003**
IL-10	0.81	ND–165.5	14.69	0.91–780.47	18.14	**0.003**
IL-11	ND	ND–ND	ND	ND–9.34	–	0.359
IL-13	1.19	0.05–2.84	9.15	ND–464.11	7.69	**<0.0001**
IL-1β	1.32	0.62–1.53	2.08	0.41–15.00	1.58	**0.0008**
IL-1rα	73.28	13.52–128.28	109.36	14.00–1,106.25	1.49	**0.011**
IL-2	ND	ND–ND	ND	ND–95.25	–	**0.015**
IL-2Rα	69.81	ND–162.12	256.38	11.14–1,372.11	3.67	**<0.0001**
IL-6	ND	ND–7.56	9.58	ND-3,788.28	–	**<0.0001**
IL-7	0.62	ND–10.57	8.74	ND–66.83	14.10	**<0.0001**
IL-8	17.92	5.26–43.15	21.95	7.37–304.59	1.22	0.229
IL-9	59.15	42.62–96.80	70.56	10.21–1,062.82	1.19	0.057
IL-12(p70)	15.91	11.09–35.49	22.37	2.18–2,269.06	1.41	0.165
IL-15	ND	ND–ND	21.44	ND–339.31	–	**0.0002**
IL-23	ND	ND–2.77	ND	ND–15.49	–	0.182
Interferon (IFN)-α2	ND	ND–2.89	43.58	ND–344.57	–	**<0.0001**
IFN-γ	30.91	17.23–34.72	60.64	ND–537.67	1.96	**0.0008**
Tumor necrosis factor-α	32.68	25.75–38.67	48.21	2.58–262.50	1.48	**<0.0001**
MIP-1α	2.50	0.98–4.96	3.53	1.19–22.42	1.41	0.091
G-CSF	17.67	9.49–24.46	39.34	13.78–504.31	2.23	**<0.0001**
Granulocyte-macrophage CSF	ND	ND–ND	62.35	ND–580.39	–	**<0.0001**
Fibroblast growth factor basic	45.15	ND–70.39	92.92	19.64–229.86	2.06	**<0.0001**
Hepatocyte growth factor	302.57	125.14–471.16	1,054.82	280.76–24,976.5	3.49	**<0.0001**
PDGF-bb	543.07	271.84–666.48	651.45	11.74–1,668.60	1.20	0.066
TGF-β1	16,764.8	4,928.0–47,560.0	33,898.0	416.00–75,620.8	2.02	**0.022**
TGF-β2	2,553.75	1,512.50–3,078.00	2,135.00	207.50–3,435.00	0.84	0.077
TGF-β3	441.60	300.00–789.60	606.00	166.00–1,554.00	1.37	0.305
IL-5	ND	ND–ND	1.45	ND–56.86	–	**0.001**
IP-10	692.68	482.47–1,392.16	928.85	359.41–34,063.25	1.34	0.114
Leukemia inhibitory factor	ND	ND–4.52	11.11	ND–893.25	–	**0.0004**
Vascular endothelial growth factor	ND	ND–13.53	97.35	ND–1,063.72	–	**<0.0001**
RANTES	11,582.3	9,480.4–14,531.4	13,749.6	680.85–22,148.46	1.19	0.103
SDF-1α	750.42	609.70–969.86	1,008.40	729.92–2,335.98	1.34	**0.0002**
Eotaxin	141.24	91.56–318.96	178.13	48.70–537.68	1.26	0.099
MIG	325.32	210.74–1,192.63	1,432.09	289.29–49,110.4	4.40	**<0.0001**
MCP-1(MCAF)	ND	ND–ND	52.82	ND–967.74	–	**<0.0001**
MIP-1β	421.48	310.31–726.69	659.13	180.24–2,207.53	1.56	**0.013**
IL-17	216.39	124.15–254.54	288.48	ND–759.86	1.33	**<0.0001**
IL-22	ND	ND–ND	ND	ND–51.06	–	0.083
IL-26	ND	ND–1.71	ND	ND–151.65	–	0.231
IL-33	0.27	ND–3.46	2.59	ND–134.16	9.59	**0.003**
Leptin male	2,200.37	583.36–4,860.99	3,082.05	186.58–89,247.8	1.40	0.139
Leptin female	7,102.62	4,005.0–10,200.2	11631.8	1,358.51–54,146.5	1.64	0.421

*MGUS and MM > HV: cytokines elevated in MGUS and MM patients vs HV; statistically significant P values are indicated in bold. Statistical analysis was performed using the Mann–Whitney U test. Leptin results were analyzed according to sex*.

*ND, not detectable (below detection level)*.

**Figure 4:**
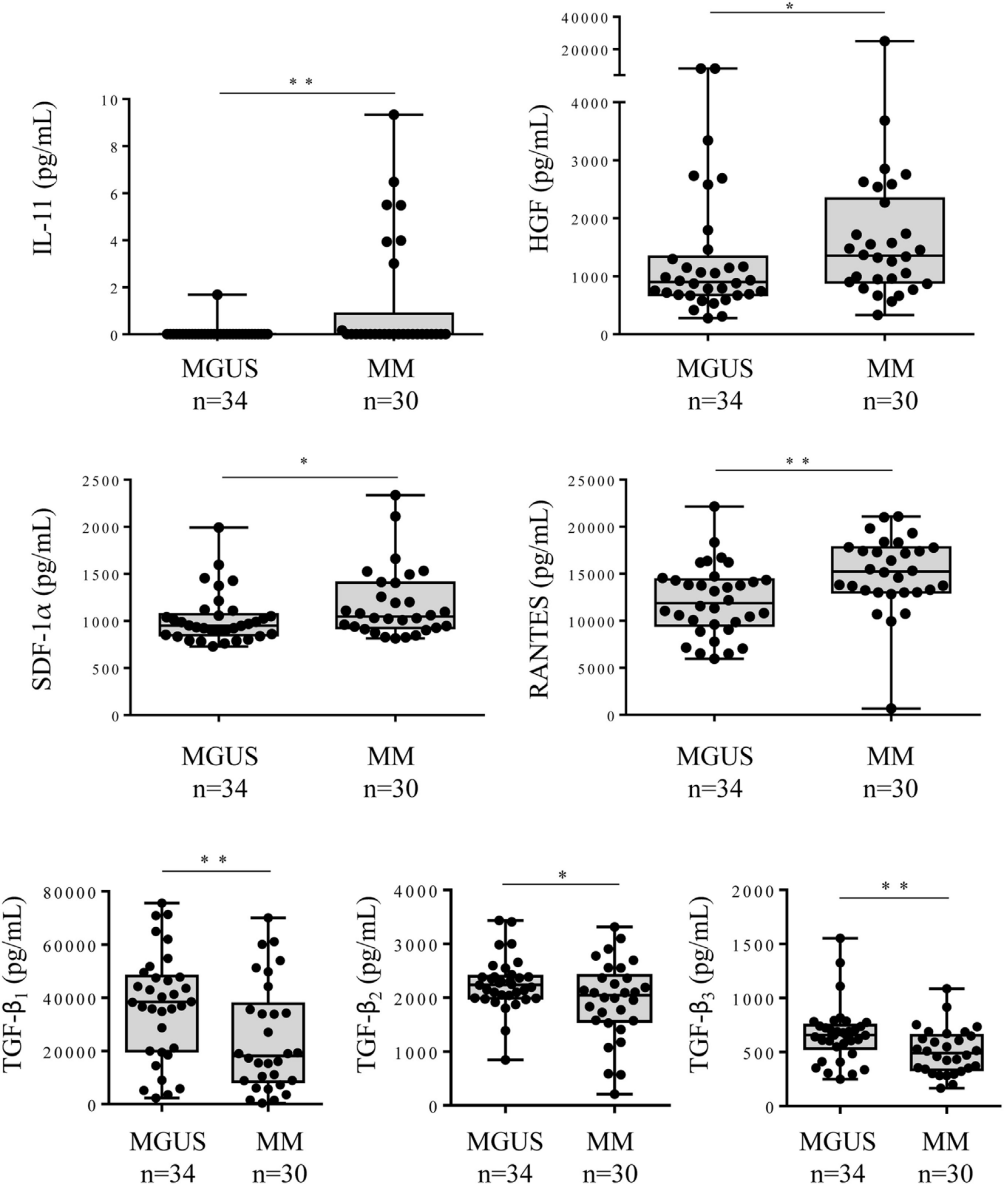
Cytokine levels in the serum of monoclonal gammopathy of undetermined significance (MGUS) and multiple myeloma (MM) patients. Forty cytokines and 2 cytokine receptors were quantified using the Biorad Luminex technology in the serum of 34 MGUS and 30 MM patients. The 6 cytokines found to be differently expressed in MGUS vs MM patients were: IL-11, HGF, SDF-1α, RANTES, TFG-β_1_, TFG-β_2_, and TGF-β_3_. Horizontal bars indicate median values ± ranges. Statistical analysis was performed using Student *t*-test. *p < 0.05 and **p < 0.01.

**Figure 5:**
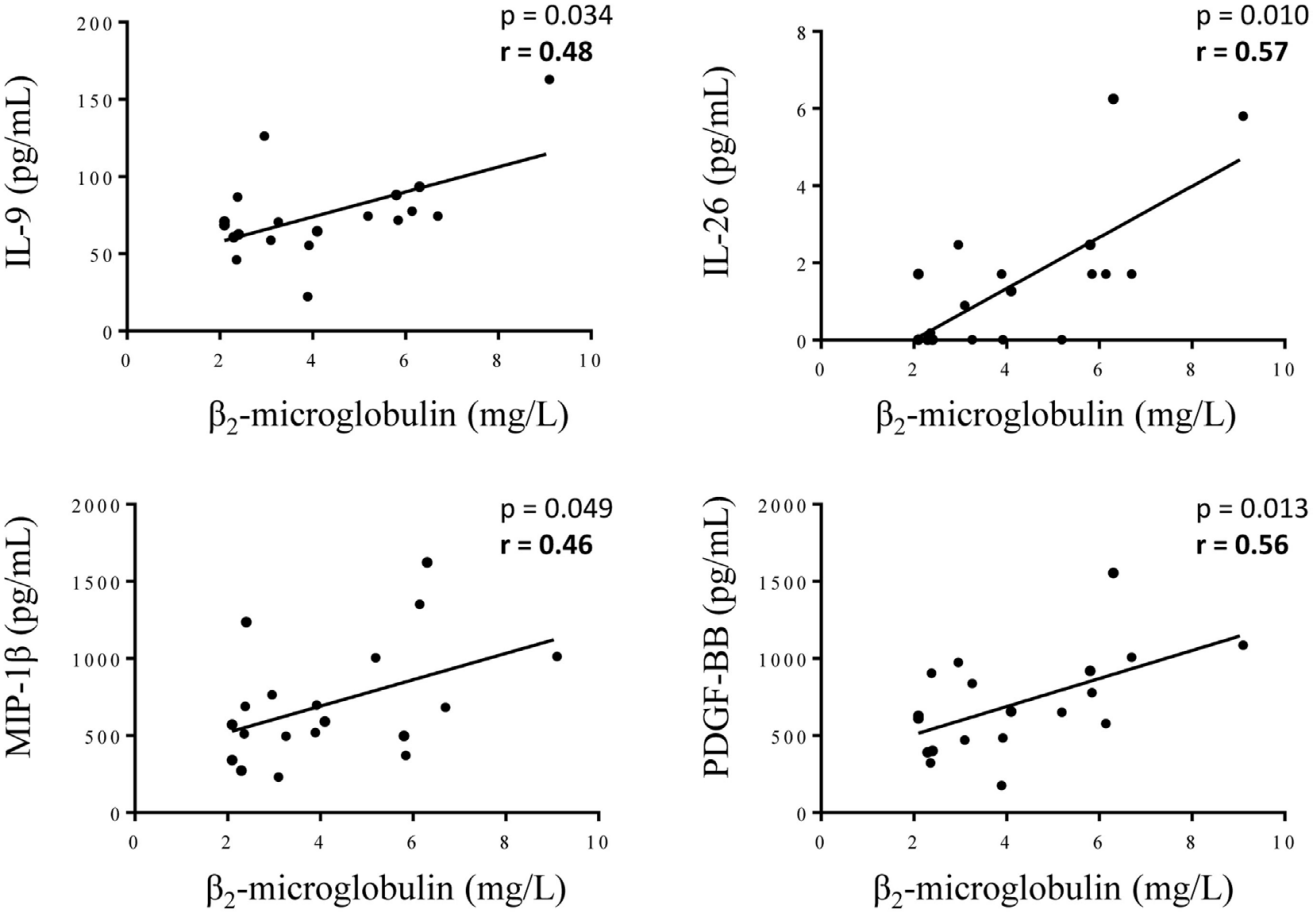
Correlations between cytokine levels and the concentration of β_2_-microglobulin in serum of multiple myeloma (MM) patients. β_2_-microglobulin concentration was found to be positively correlated with the concentration of IL-9, IL-26, MIP-1β, and PDGF-BB for the 19 MM patients with available β_2_-microglobulin data. Statistical analysis was performed using the Spearman *t*-test.

The comparison between the MGUS and SM/MM groups (Figure 4; Table S1 in Supplementary Material) revealed that only four molecules were slightly but significantly higher in SM/MM patients: HGF, IL-11, SDF-1α, and RANTES. Interestingly, HGF is a survival factor which exerts a pro-tumoral and both pro- and anti-inflammatory action. TGF-β_1_, TGF-β_2_, and TGF-β_3_, known for their anti-proliferative and pro-differentiation effect on hematopoietic cells, were more expressed in MGUS than in SM/MM. Since high levels of HGF are considered of poor prognosis in MM, we explored eventual correlations between cytokine levels and β_2_-microglobulin, an important biomarker in the prognosis of MM. Figure 5 shows the positive correlations found between β_2_-microglobulin concentration and serum levels of IL-9, IL-26, MIP-1β (pro-inflammatory molecules), and PDGF-BB. Of note, IL-26 is involved in anti-microbial immunity.

### IgG Hyposialylation, Pro-inflammatory Status, and Disease Severity

**Figure 6:**
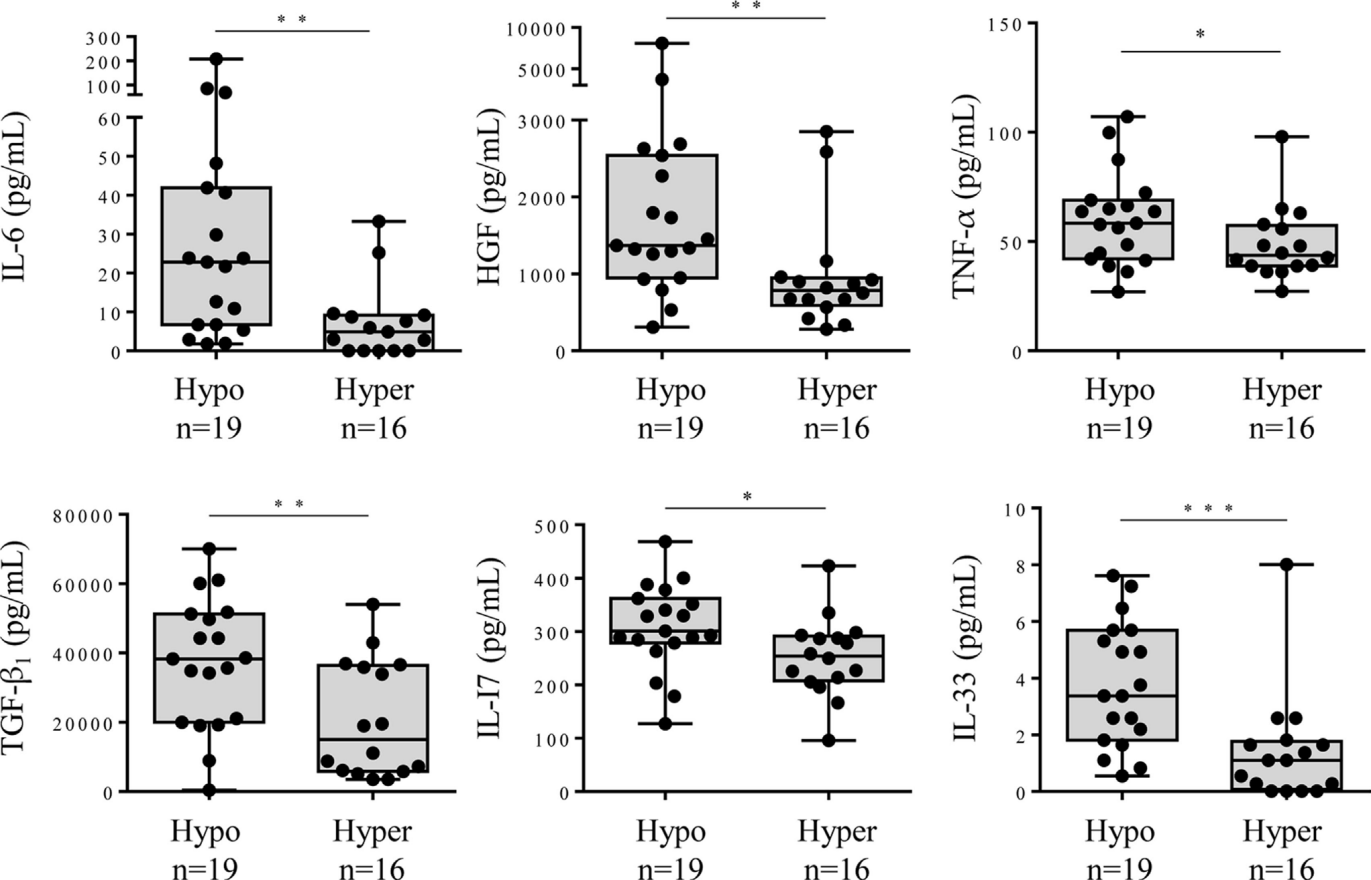
Cytokine levels in the serum of monoclonal gammopathy of undetermined significance (MGUS) and smoldering myeloma (SM)/Multiple myeloma (MM) patients with hyposialylated or hyper-sialylated pc IgGs. Forty cytokines and 2 cytokine receptors were quantified using the Biorad Luminex technology in the serum of 19 MGUS or SM/MM patients with hyposialylated pc IgGs (hypo), and 16 MGUS or SM/MM patients with hyper-sialylated pc IgGs (hyper). The 6 cytokines found to be more expressed in patients with hyposialylated pcIgGs were interleukin 6 (IL-6), hepatocyte growth factor (HGF), tumor necrosis factor (TNF)-α, TGF-β_1_, IL-17, and IL-33. Horizontal bars indicate median values ± ranges. Statistical analysis was performed using Mann–Whitney *U* test. *p < 0.05, **p < 0.01, and ***p < 0.001. Normal values for IL-6: <9 pg/mL; HGF, median: 195 pg/mL, range: 63–1,283 pg/mL; TNF-α, median: 0 pg/mL, range: 6–98 pg/mL; TGF-β_1_, median: 47 pg/mL, range: 0–932 pg/mL; IL-17, median: 0 pg/mL, range: 0.22–31 pg/mL; and IL-33, not defined.

**Figure 7:**
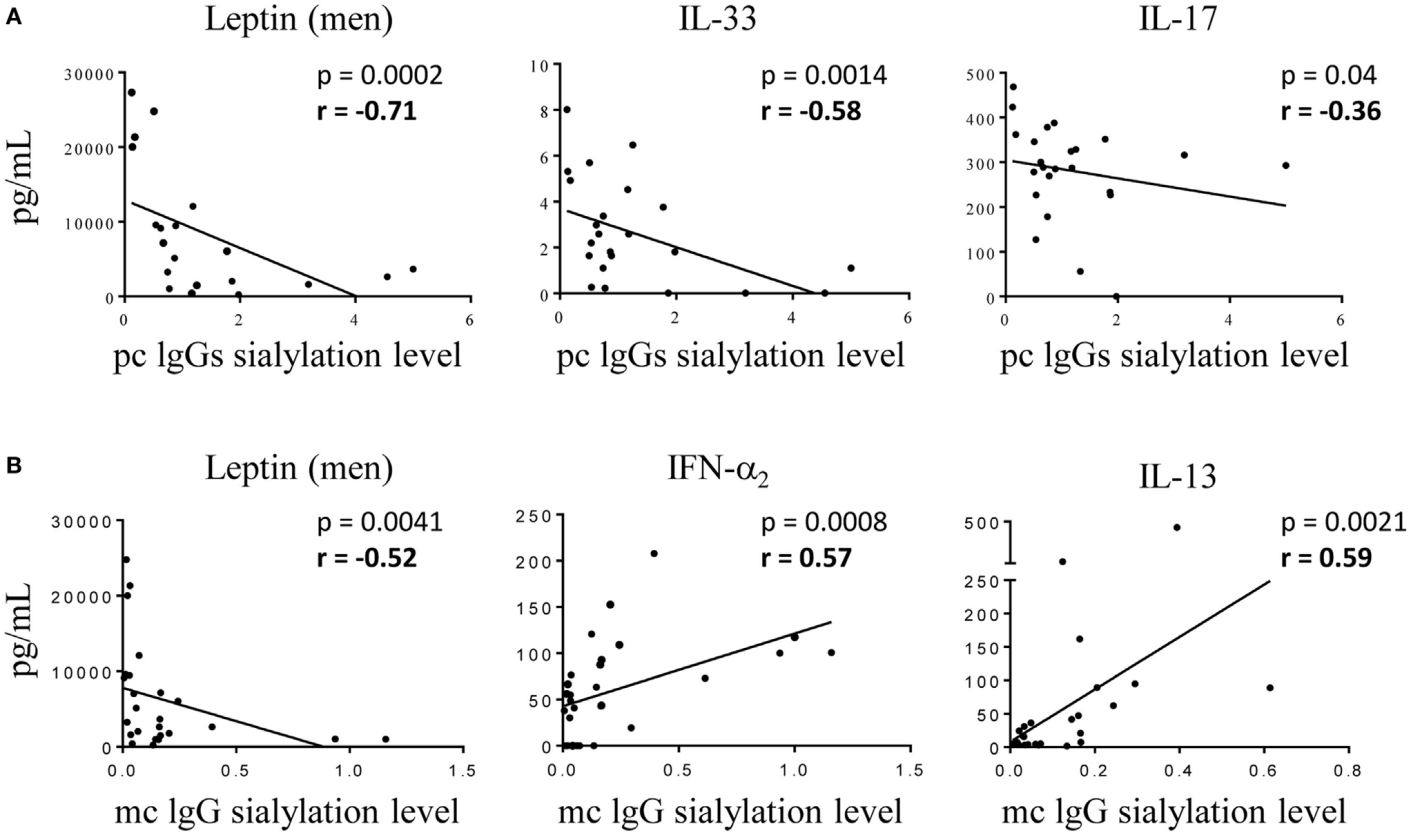
Correlations between cytokine levels in serum of multiple myeloma (MM) patients and level of IgG sialylation. **(A)** Pc IgGs sialylation level and the concentration of leptin (men only), IL-33, and IL-17 were negatively correlated (*n* = 25 MM patients with available pc IgG sialylation data). **(B)** Mc IgG sialylation level was negatively correlated with leptin (men only), and positively correlated with the concentration of interferon (IFN)-α_2_ and IL-13 (*n* = 30 MM patients with available mc IgG sialylation data). Statistical analysis was performed using the Spearman *t*-test.

Hyposialylation of IgGs has been described as a hallmark of pro-inflammatory state in pathologic contexts other than MM. We analyzed cytokine levels in the serum of MGUS, SM, and MM patients according to the sialylation level of their pc IgGs. Figure 6 shows that patients presenting with hyposialylated pc IgGs have significantly higher levels of major pro-inflammatory cytokines (IL-6, TNF-α, TGF-β_1_), and also have higher levels of HGF, IL-17, and IL-33, compared to patients with hyper-sialylated pc IgGs (*p* < 0.05). These results were confirmed when data were analyzed for potential correlations between cytokine levels and the degree of sialylation of pc IgGs or purified mc IgGs. In SM/MM group, the degree of sialylation of pc IgGs was negatively correlated with levels of IL-17 and IL-33, and also with the concentration of leptin (men only) (Figure 7A). The degree of sialylation of purified mc IgGs was also negatively correlated with leptin (men only) (Figure 7B), and positively correlated with the levels of IFN-α_2_ and IL-13. Thus, in MM, sialylation of pc IgGs is inversely correlated with the levels of IL-17 and IL-33, two cytokines important for anti-microbial response. The sialylation level of purified mc IgGs increased with the level of 2 anti-inflammatory cytokines: IFN-α_2_ and IL-13.

**Figure 8:**
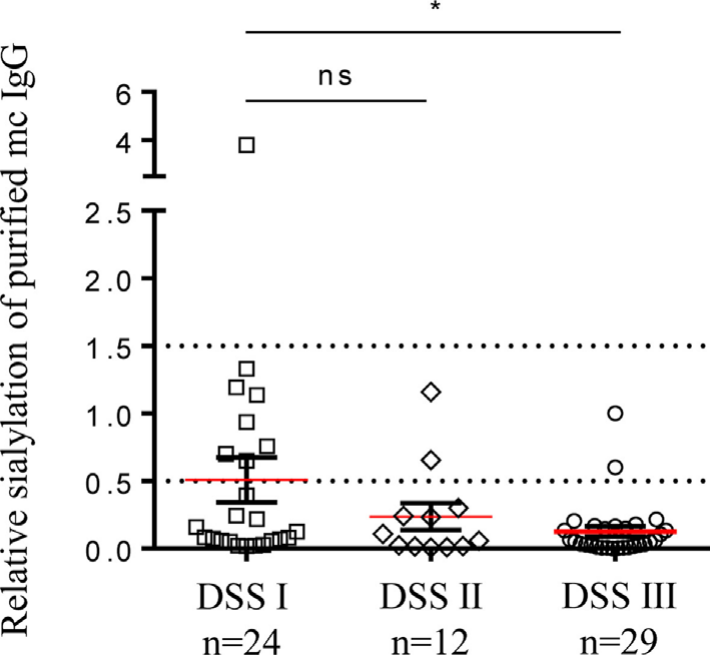
Sialylation level of mc IgG from patients diagnosed with multiple myeloma (MM) at Durie–Salmon Staging (DSS) I, II, or III. Sialylation level of mc IgGs was compared among groups of MM patients with DSS stage I, II, or III. Horizontal bars indicate mean ± SEM. Statistical analysis was performed using Kruskall–Wallis test followed by Dunn’s Multiple Comparison test. *p < 0.05.

In addition, we found that sialylation of purified mc IgGs was significantly lower for MM patients with advanced disease—Durie–Salmon staging (DSS) III—than for those with DSS I (Figure 8). Similarly, C-reactive protein (CRP) concentration, a common inflammation marker, was inversely correlated with the sialylation level of pc IgGs (MGUS, SM, and MM patients, Figure S3 in Supplementary Material), and the β_2_-microglobulin concentration was inversely correlated with the sialylation level of purified mc IgGs (MGUS, SM, and MM patients, Figure S3 in Supplementary Material). Altogether, these findings suggest that mc IgG hyposialylation could be a new marker of disease severity in MM.

## Discussion

There is increasing evidence that chronic Ag stimulation, Ag-driven selection of plasma cell clones and subsequent mc IgG production, initiate MGUS and MM for subsets of patients. Nair et al. identified lyso-glucosylceramide (LGL1) as a potential target of mc Ig of MGUS and MM patients (43). We recently reported that six infectious pathogens, including carcinogenic viruses (EBV, HCV, and HSV) and bacteria (*H. pylori*), are the targets of ~23% of purified mc IgGs from MGUS, SM, and MM patients (11). Thus, for a significant percentage of patients, MGUS may result from chronic infectious Ag-driven clone proliferation and abnormal immune response, either to self-proteins or to infectious pathogens. Over time, the chronically stimulated lineage is at increased risk of genetic alteration and subsequent malignant transformation and overt MM. Several studies demonstrated that efficient anti-viral activity is associated with a dramatic shift in the IgG-glycosylation profile toward agalactosylated, afucosylated, and asialylated glycans (20, 44). Here, we demonstrate that the purified mc IgGs from MGUS, SM, and MM patients exhibit a very low level of sialylation in comparison to pc IgGs from HVs and pc IgGs from the same MGUS, SM, and MM patients. Furthermore, purified mc IgGs from MM patients were less sialylated than mc IgGs from MGUS patients, and purified mc IgGs targeting specifically an infectious pathogen had an even lower sialylation status than mc IgGs not directed against an infectious pathogen of the MIAA test. Moreover, hyposialylation of purified mc IgGs was concomitant with increased levels of cytokines that played a major role in inflammation and anti-microbial response. These results suggest that infection, inflammation, and an abnormal immune response are early events in a subset of MGUS and MM.

At least two main questions arise from these findings. The first concerns the molecular mechanisms that lead to hyposialylation of mc IgGs, and the link with inflammation. The second concerns the functional consequences of IgG hyposialylation on both the activation of macrophages and their subsequent production of pro- or anti-inflammatory cytokines, and on the Ab function of hyposialylated mc IgGs, especially when they target a pathogen.

Regarding the links between mc IgG hyposialylation and inflammation in chronic hematological malignancies, our results show that the inflammatory environment in MGUS and in MM is associated with the production of poorly sialylated mc IgG. Yet it is unlikely that alone, inflammation is sufficient to explain hyposialylation of mc IgGs in all cases, or all pc IgGs would be hyposialylated in MM. Hyposialylation of pc IgGs is observed in MM, but for only 11% of patients. Similarly, pc IgGs of MPN patients were normally sialylated, although chronic inflammation is more severe in MPNs than in MGUS and MM. Analysis of the expression and/or activity of the α2,6-sialyltransferase and sialidase enzymes in clonal plasma cells may be more informative to understand the potential mechanisms leading to hyposialylation of mc IgG. Indeed, Oefner et al. (33) showed that the stimulation with T-cell-dependent Ag under inflammatory conditions can result in the production of plasma cells that express low levels of α2,6-sialyltransferase and secrete desialylated IgGs. Fc sialylation is, thus, crucial for the differentiation between a tolerogenic immune status and a pathogenic immune status, the latter being directly correlated to the α2,6-sialyltransferase activity of plasma cells (33). Similar results were observed in T-cell acute lymphoblastic leukemia, where a decreased sialylation of membrane proteins was directly correlated with α2,6-sialyltransferase mRNA expression and activity (45). Furthermore, low IgG sialylation due to increased sialidase activity has been described in various cancers (46, 47). Unfortunately, we did not have access to patient plasma cells in the present study and the activity of these enzymes could not be investigated.

When inflammation was associated with specific viral Ag-specificity of the mc IgG, further decrease in the sialylation level of mc IgGs was observed in MM, especially for patients in DSS stage III (Figure 8). These observations are consistent with a deleterious effect of the hyposialylation of mc IgGs in MM disease evolution. It is established that the sialylation of IgGs dramatically changes their physiologic role, converting IgG from pro-inflammatory into anti-inflammatory (18, 26). The small fraction of sialylated IgGs is responsible for the immunosuppressive activity of IVIg (16). Recently, Barrios et al. (48) showed that the sialylation level of IgG structures decreased in patients with chronic kidney disease, thus demonstrating that sialylated glycans play an independent protective role in chronic kidney disease. Moreover, we recently demonstrated that the sialylation of the anti-donor specific Ab against HLA class I are more sialylated in kidney transplant recipients who do not develop Ab-mediated rejection, than in patients who develop Ab-mediated rejection (40). Also, Quast et al. (28) demonstrated that the increase of the Fc IgG sialylation impairs the CDC due the inhibition to C1q binding. In fact, crystallographic and biophysical studies of sialylated and asialylated IgG Fc fragments showed that IgG Fc sialylation leads to conformational changes in the protein (49, 50).

The initial discovery that IgG sialylation plays a key role in the suppressive activity of IVIg in autoimmunity was a hallmark in the appreciation of the role of glycans in immune responses (16–18, 27). In murine models, sialylated IgGs bind to DC-SIGN, inducing the production of IL-33, an infection-linked cytokine that activates basophils to produce IL-4, leading to the upregulation of the inhibitory Fc receptor FcγRIIb (27, 51). In our study, the IL-33 and IL-4 levels were not different between MGUS and MM patients (Figure 4; Table S1 in Supplementary Material), thus in MM the IL-33 level may not be sufficient to induce IL-4 and expression of FcγRIIb. In fact, Musolino et al. (52) found that the IL-33 plasma levels were reduced in MM and were associated with more advanced disease. These observations support the hypothesis that latent infection and inflammation could be the early events for subsets of MGUS and MM, and *via* their pro-inflammatory effects, the hyposialylated IgGs (mc IgGs and in 11% of cases, pc IgGs also) contribute to the inflammatory environment and the progression to myeloma.

Our study shows that inflammation occurs early in myeloma pathogenesis since a very similar chronic state of inflammation was observed in MGUS and MM patients *vs* healthy controls: 35/42 molecules linked to inflammation were similarly increased in MGUS and MM. The similar inflammatory status observed in MGUS, considered a benign condition, and MM, a severe, overtly malignant and often invalidating disease, was unexpected. A such observation was observed by Zheng et al. (53) who showed that only IL-17 was highly increased in MM patients in comparison to MGUS patients but their cohort included 55 MM patients and only 8 MGUS patients. HGF was more strongly expressed in MM. HGF plays an important role in MM, inducing IL-11 and IL-6, two markers of disease activity and poor prognosis in MM (54–56). HGF and IL-11 are anti-inflammatory cytokines; in addition, IL-11 promotes bone destruction by osteoclasts and inhibits bone formation by osteoblasts, thus causing cancer-induced bone lesions (57). We also confirmed that TGF-β is less expressed in MM than in MGUS (58). This could be explained by the anti-proliferative and pro-differentiation effects on hematopoiesis of TGF-β *in vivo* (59).

In the context of MM, we found positive correlations with β_2_-microglobulin concentration and expression levels of IL-9, IL-26, MIP-1β and PDGF-BB (Figure 5). β_2_-Microglobulin helps to characterize the severity and define the stage and prognosis of MM. Like MIP-1α, MIP-1β plays a role in hematopoiesis and osteoclast recruitment, and MIP-1α/β secretion correlates with lytic bone lesions in MM patients (60, 61). Several cytokines, including VEGF and PDGF-BB, are released by MM tumoral cells and also by endothelial cells, thereby contributing to the marked bone marrow micro-vessel density, a constant hallmark of active MM and of acquired refractoriness of MM plasma cells to conventional therapies (62, 63). Consistently, anti-tumor/vessel dasatinib, an inhibitor of PDGF receptor, significantly delays MM plasma cell growth and angiogenesis *in vivo* (64).

Since IgG hyposialylation is a hallmark of pro-inflammatory state, we investigated whether sialylation of IgG was linked to the secretion of specific pro-inflammatory cytokines. Such studies had never been done previously. We found that patients who have hyposialylated pc IgGs in addition to mc IgGs secrete several pro-inflammatory cytokines (IL-6, TNF-α, TGF-β, IL-17, and IL-33) or cytokines involved in MM progression (HGF) at significantly higher levels than patients with hyper-sialylated pc IgGs. The increased production of IL-17 may be of interest: in addition to its anti-microbial action, inhibition of Th1 response, and production of pc IgM and IgA, IL-17 has been shown to act directly on the expression of α2,6-sialyltransferase. Moreover, this enzyme is downregulated by IL-17 in autoimmune diseases, leading to decreased IgG sialylation (65). Accordingly, we found an inverse correlation between levels of IL-17 (and IL-33) and the degree of sialylation of pc IgGs in both MM and MGUS. Conversely, the levels of anti-inflammatory cytokines IFN-α_2_ and IL-13 were positively correlated with mc IgG sialylation. We also found an inverse correlation between the sialylation level of both pc and mc IgG and the leptin level in male patients. An increase in leptin level in serum has been observed in blood malignancies (66, 67) and particularly in newly diagnosed MM (68). This adipokine induces pro-inflammatory IL-1β as well as the expression of IL-6, TNF-α and many genes involved in the growth and metabolism of MM plasma cells (68, 69).

**Figure 9:**
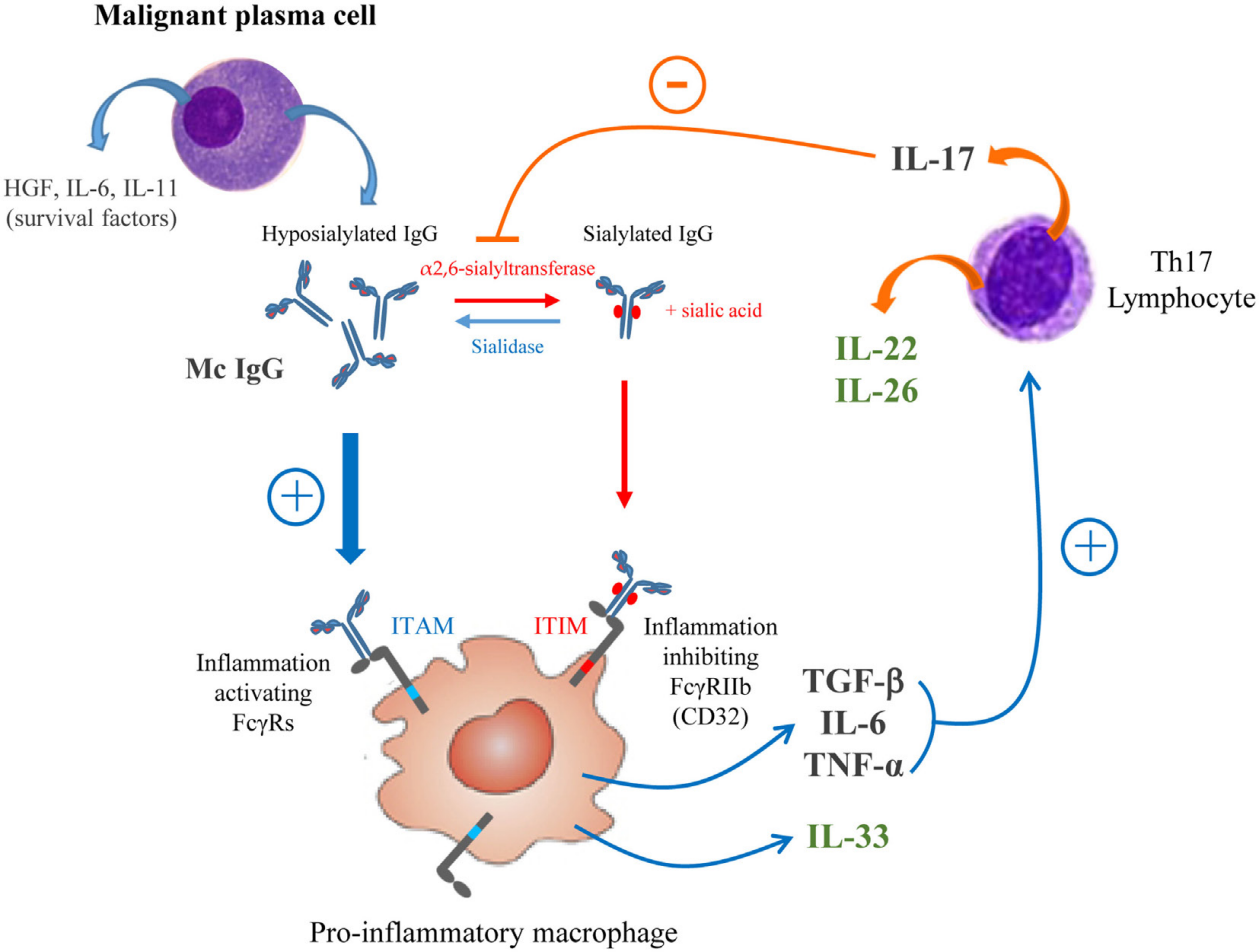
Schematic model of the regulation loop linking inflammation and IgG sialylation in multiple myeloma (MM). A pro-inflammatory microenvironment induces the secretion by malignant plasma cells of high amount of hyposialylated mc IgG which preferentially bind to activating receptors FcγRs (ITAM, blue pathway). To the contrary, sialylated IgGs preferentially bind to the inhibiting receptor FcγRIIb (ITIM, red pathway). The activation of macrophages by hyposialylated IgGs leads to the secretion of pro-inflammatory cytokines TGF-β, interleukin 6 (IL-6), tumor necrosis factor (TNF)-α and IL-33. TGF-β, IL-6, and TNF-α are known to stimulate the expression of IL-17 by Th17-lymphocytes, which are elevated in MM. IL-17 may inhibit the expression of the α2,6-sialyltransferase thus contributing to decrease IgG sialylation. MM patients with pathogen-specific mc IgG presented with the less hyposialylated mc IgG and highest level of IL-22, IL-26 and IL-33 (green cytokines) suggesting that this regulation loop may be amplified.

On the basis of our previous (11) and present findings, we propose a schematic model of the relation between chronic inflammation/infection and the structure/function of mc IgG in myeloma (Figure 9). Over-expression of HGF is observed in MM vs MGUS, as well as IL-22, IL-26, and IL-33 in MM patients with a pathogen-specific mc IgG (MIAA+) vs MM patients with mc IgG of undetermined specificity, is consistent with the presence of a pro-inflammatory microenvironment. Under these conditions, MM plasma cells produce large quantities of hyposialylated mc IgGs, which activate macrophages *via* FcγRs. The resulting secretion by activated macrophages of TGF-β_1_, TNF-α, and IL-6 stimulates the production of Th17 cells, which secrete IL-17, IL-22, and IL-26. IL-22 is a marker of poor prognosis in MM (70), and IL-17 can induce the downregulation of sialyltransferase activity, thus maintaining the hyposialylation of mc IgG and to a lesser extent, of pc IgGs too (33).

## Conclusion

In MGUS, SM, and MM, mc IgGs are hyposialylated compared to pc IgGs from both healthy controls and MPN patients. Mc IgG hyposialylation was lowest in MM, particularly when the purified mc IgG targeted an infectious pathogen. Although the exact mechanisms of IgG hyposialylation in MM remain to be identified, IgG hyposialylation correlated with the overproduction of several cytokines (IL-17, IFN-α_2_, IL-33, and IL-13) that play a major role in inflammation and anti-microbial response. Altogether, the data suggest that infection, inflammation, and an abnormal immune response are early events for subsets of MM patients.

## Ethics Statement

The study was performed with the approval of the local ethics committee (# RC12 0085, University Hospital of Nantes) and the Commission Nationale de l’Informatique et des Libertés (CNIL # 912335).

## Author Contributions

JH, SH, AB, and EB-C designed the research, analyzed data, and wrote the paper. AB, JH, SA-M, NM, and HP performed experiments. AT, CR, DC, LG, PM, EP, and FG contributed patient samples and data, and critically read the manuscript. AN contributed to the statistical analysis of data. HP, AN, and SB critically read the manuscript. All authors gave final approval of the version to be submitted to publication and agreed to be accountable for all aspects of the work in ensuring that questions related to the accuracy or integrity of any part of the article are appropriately investigated and resolved.

## Conflict of Interest Statement

The authors declare that the research was conducted in the absence of any commercial or financial relationships that could be construed as potential conflicts of interest.
