# Supplementary material for: Pro-inflammatory State in Monoclonal Gammopathy of Undetermined Significance and in Multiple Myeloma Is Characterized by Low Sialylation of Pathogen-Specific and Other Monoclonal Immunoglobulins
Source: Front Immunol. 2017 Oct 19;8:1347. doi: 10.3389/fimmu.2017.01347 (PMC5653692; doi:10.3389/fimmu.2017.01347)
Supplement: Supplementary file 1 [file table_1.pdf]

Supplementary Table 1: Inflammation-linked molecules altered in MM compared to MGUS.

Cytokines	MGUS (n=34)		MM (n=30)		MM > MGUS		MM < MGUS	
	median	range	median	range	fold change	*P value*	fold change	*P value*
IL-11	ND	ND-1.69	ND	ND-9.34	-	**0.004**		
HGF	902.61	280.76-8129.28	1354.14	333.17-24973.5	1.50	**0.039**		
TGF-β1	38442.6	2304.0-75620.8	18150.4	416.0-70080.0			2.12	**0,001**
TGF-β2	2240.75	843.75-3435.00	2045.75	207.50-3312.50			1.10	**0.024**
TGF-β3	658.20	248.00-1554.00	490.80	166.00-1086.00			1.34	**0,005**
RANTES	11888.3	5978.6-22148.5	15240.1	680.8-21091.8	1.28	**0.001**		
SDF-1α	950.58	729.92-1991.96	1046.91	816.02-2335.98	1.10	**0.042**		

MM>MGUS: level in serum elevated in MM compared to MGUS; MM<MGUS: level in serum reduced in MM compared to MGUS. Statistical analysis was performed using the *t*-test. Significant differences are shown in bold. ND: not detectable.
