# Supplementary material for: Pro-inflammatory State in Monoclonal Gammopathy of Undetermined Significance and in Multiple Myeloma Is Characterized by Low Sialylation of Pathogen-Specific and Other Monoclonal Immunoglobulins
Source: Front Immunol. 2017 Oct 19;8:1347. doi: 10.3389/fimmu.2017.01347 (PMC5653692; doi:10.3389/fimmu.2017.01347)
Supplement: Supplementary file 2 [file image_1.pdf]

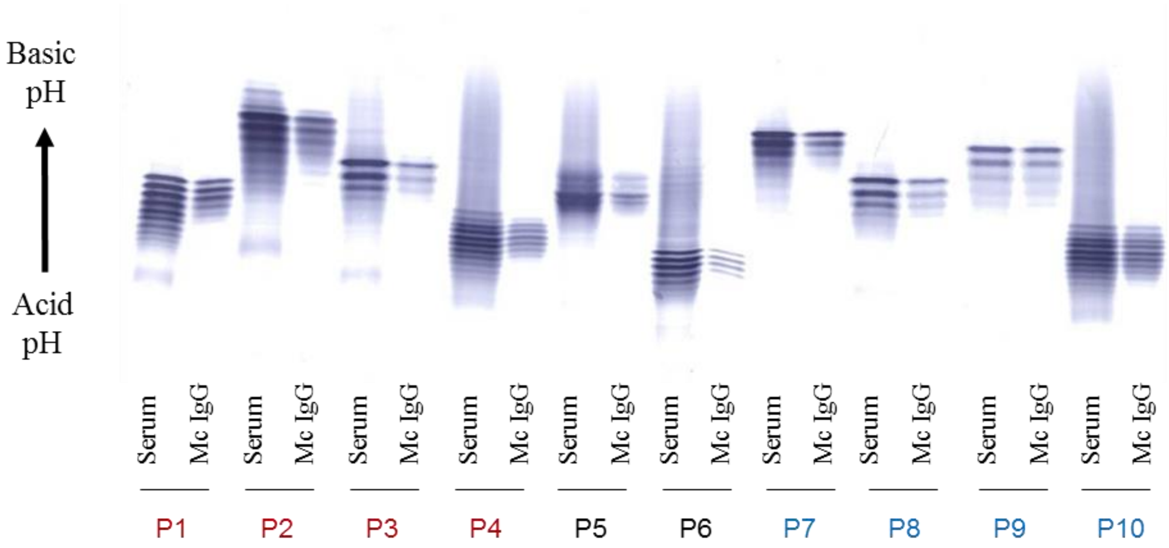

Supplementary Figure 1: Purity analysis of purified mc IgGs by Isoelectro-focusing (IEF) and immuno Blot. For each patient, serum and purified mc IgG from the serum were submitted to isoelectrofocalisation gel electrophoresis followed by immunoblotting using a HRP-anti-human IgG-γ chain antibody (HRP anti-human IgG Ab). Examples of 10 patients (P1 to P10). MGUS are shown in blue; smoldering myeloma (SM) are shown in black, and MM are shown in red.
