# Supplementary material for: Pro-inflammatory State in Monoclonal Gammopathy of Undetermined Significance and in Multiple Myeloma Is Characterized by Low Sialylation of Pathogen-Specific and Other Monoclonal Immunoglobulins
Source: Front Immunol. 2017 Oct 19;8:1347. doi: 10.3389/fimmu.2017.01347 (PMC5653692; doi:10.3389/fimmu.2017.01347)
Supplement: Supplementary file 3 [file image_2.pdf]

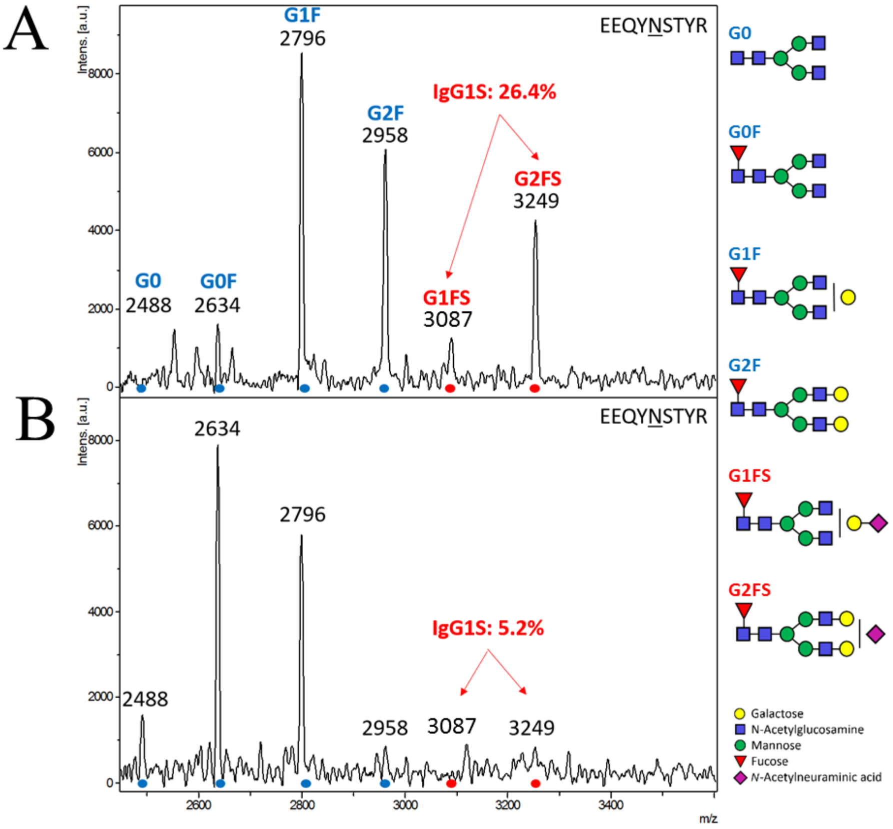

Supplementary Figure 2: Analysis of glycoforms of two purified mc IgGs by mass spectrometry. A highly sialylated purified mc IgG1 of a MGUS patient **(A)** and a weakly sialylated purified mc IgG1 of a MGUS patient **(B)** were studied by mass spectrometry. Sialylation glycoforms G1FS and G2FS at m/z 3087 and 3249 respectively are indicated with red points. Non-sialylated glycoforms are indicated with blue points. The percentage of sialylation is the ratio of the area of the sialylated peaks divided by the area of total peaks.
