# Supplementary material for: Pro-inflammatory State in Monoclonal Gammopathy of Undetermined Significance and in Multiple Myeloma Is Characterized by Low Sialylation of Pathogen-Specific and Other Monoclonal Immunoglobulins
Source: Front Immunol. 2017 Oct 19;8:1347. doi: 10.3389/fimmu.2017.01347 (PMC5653692; doi:10.3389/fimmu.2017.01347)
Supplement: Supplementary file 4 [file image_3.pdf]

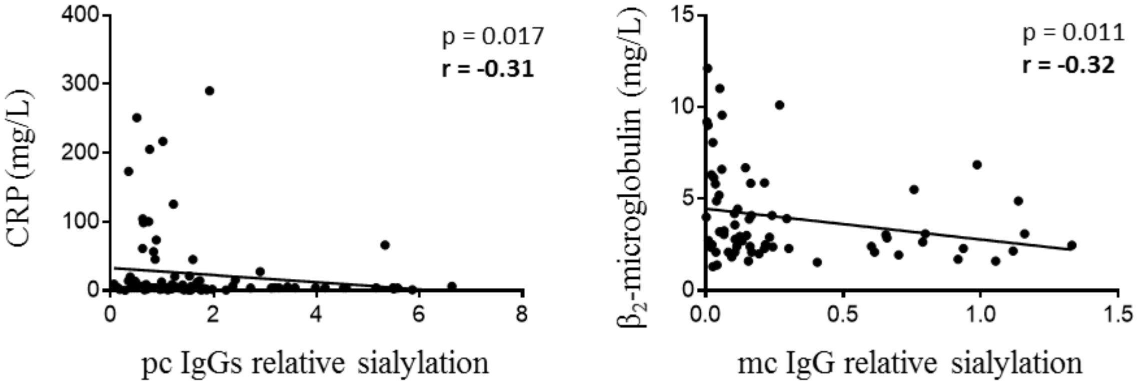


**Supplementary Figure 3: Correlations between C-reactive protein, $\beta_2$-microglobulin and sialylation of pc IgGs or mc IgGs from MGUS, SM and MM patients.** C-reactive protein (CRP) and $\beta_2$-microglobulin concentrations were available for 97 and 69 patients, respectively (MGUS, SM and MM patients studied together). The CRP concentration was negatively correlated with the sialylation level of pc IgGs, and the $\beta_2$-microglobulin concentration was negatively correlated with the sialylation level of mc IgGs. Statistical analysis was performed using the Spearman *t*-test.
